# Infection of Tissue Expander With Candida Parapsilosis

**Published:** 2012-05-22

**Authors:** Eran Lavi, Allan Billig, Dalit Amar, Rami Neuman, Alex Margulis, Tomer Tzur

**Affiliations:** Department of Plastic and Reconstructive Surgery, Hadassah Medical Center, Hebrew University, Jerusalem, Israel

## DESCRIPTION

**Figure 1:**
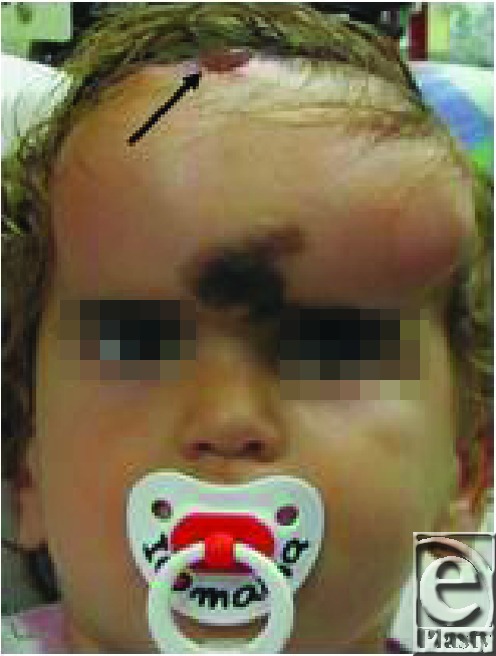
Infection of the tissue expander pocket with Candida Parapsilosis. The arrow indicates the dehiscence in the surgical wound.

A 13-month-old boy underwent placement of a tissue expander in the subgaleal plane in the forehead. The indication for skin expansion was a large congenital melanocytic nevus involving the forehead and glabella. Serial inflations of the expander were performed in the plastic surgery clinic using sterile equipment. Alcohol was used to prepare the skin overlying the injection port. Three months following the insertion of the tissue expander, the patient presented to the emergency department with fever up to 102.2°F (39°C), and a yellowish discharge from the surgical wound. The wound was dehisced (Fig 1).

Cultures taken from the wound revealed growth of Candida Parapsilosis in 3 different sets taken 24 hours apart.

## QUESTIONS

1. **What is the most common complication of tissue expansion in children?**
2. **What are the most common pathogens causing infections of tissue expanders?**
3. **What are the keys for a successful treatment of tissue-expander infections?**

## DISCUSSION

Infection is the most common complication of tissue expansion in children with an incidence ranging between 4.4% and 10% in different series.^1^ It is often secondary to introduction of organisms at the time of tissue expander insertion or due to hematogenous seeding of the expander by bacteria from distant sites (ie, acute otitis media). Common pathogens reported are normal skin flora pathogens (*Staphylococcal* and *Streptococcal* species) and *Pseudomonas aeruginosa*.

A review of the literature reveals several reports of fungal contamination in breast implants. Most of these reports describe infection of the fluid inside the lumen of inflatable breast implants. In 1975, Truppman et al^2^ described 4 cases of fungal infection in breast implants with *Candida albicans* in one patient, and a *Curvularia* species in another patient. In the remaining 2 cases reported in this article, no fungal cultures were obtained, but the solution filling the implant contained a black particulate material, with septated hyphae. In 1983, Williams et al^3^ reported intraluminal infections of breast saline impants with *Aspergillus niger*. In 1995, Young et al^4^ demonstrated contamination of a saline breast implant with *Paecilomyces variotii*.

*Candida parapsilosis* is an emerging major human pathogen that has dramatically increased in prevalence and significance over the past 2 decades. Today, it is one of the leading causes of invasive candidal disease. *Candida parapsilosis* is typically a commensal organism of the human skin, and its pathogenicity is limited by an intact integument. *Candida parapsilosis* infections are usually associated with prosthetic devices and indwelling catheters. Nosocomial spread of the pathogen through the hands of health care workers is the common vector. It was also described in nursing mothers as a cause of infantile oropharingeal candidiasis.^5^

**Figure 2:**
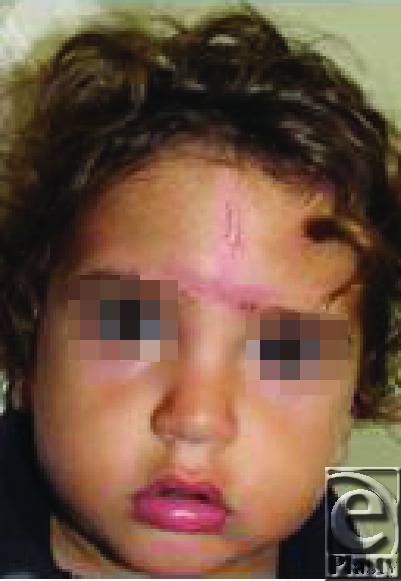
Postoperative views after removal of the expander, excision of the nevus, and reconstruction with an expanded flap.

In this report, we present a child with an infection of the tissue-expander pocket with this pathogen. After 6 days of treatment with intravenous fluconazole, the patient was operated and the tissue expander was removed. Excision of the nevus and reconstruction with an expanded skin flap were conducted as planned. The postoperative course was uneventful and the patient was discharged from the hospital on the second postoperative day with oral fluconazole for additional 2 weeks (Fig 2).

The keys to his successful treatment were prompt diagnosis, culture-targeted antifungal therapy, and removal of the foreign body. Using these principles, a successful excision of the nevus and reconstruction with an expanded flap were still possible despite of the acute infection.

## References

[1] Adler N, Dorafshar Amir H (2009). Tissue expander infection in pediatric patients: management and outcomes. Plast Reconstr Surg.

[2] Truppman ES, Ellenby JD, Schwartz BM (1979). Fungi in and around implants after augmentation mammoplasty. Plast Reconstr Surg.

[3] Williams K, Walton RL, Bunkis J (1983). Aspergillus colonization associated with bilateral silicone mammary implants. Plast Reconstr Surg.

[4] Young LV, Hertl C, Murray PR (1996). Paecilomyces variotii contamination in the lumen of a saline-filled breast implant. Plast Reconstr Surg.

[5] Trofa D, Gacser A, Nsanchuk JD (2008). Candida parapsilosis, an emerging fungal pathogen. Clin Micrib Rev.

